# Comparison of Spatio‐Temporal Dynamics and Composition in Size‐Fractionated and Unfractionated Northwestern Atlantic Microbial Communities

**DOI:** 10.1111/1758-2229.70206

**Published:** 2026-02-06

**Authors:** Diana Haider, Jennifer Tolman, Robert G. Beiko, Julie LaRoche

**Affiliations:** ^1^ Department of Biology Dalhousie University Halifax Nova Scotia Canada; ^2^ Faculty of Computer Science Dalhousie University Halifax Nova Scotia Canada; ^3^ Institute for Comparative Genomics Dalhousie University Halifax Nova Scotia Canada

**Keywords:** amplicon sequencing, filtration methodology, microbial community, size fractionation

## Abstract

Size fractionation, filtering water sequentially through a large (3 μm) and fine (0.2 μm) pore size filter, is a widely applied approach to target specific microbial size ranges, differentiating between particle‐associated, and free‐living microorganisms. To characterise its impact on microbial diversity and its comparability to unfractionated samples, we analysed 16 weekly ocean samples across five depths during a phytoplankton spring bloom. We used a universal marker to characterise prokaryotes, eukaryotes and chloroplasts in unfractionated (> 0.2 μm), fractionated (large > 3 μm, small 0.2–3 μm) and de‐fractionated samples, the reconstitution of the small and large fractions. The particle‐associated fraction was the most different community from all other samples, and de‐fractionating before or after sequencing results in a community that is most similar to unfractionated samples in terms of composition and richness with the exception of very rare taxa. Across all depths and weeks, 75%–97% of ASVs were shared, but some discrepancies in relative abundances were unresolved, including for some lineages of free‐living Bacteroidota. Community composition differences from size fractionation were more pronounced during the bloom period in comparison to pre‐bloom. Differential‐abundance analysis detected at most one significantly different ASV between fractionated and de‐fractionated samples, highlighting the similarity in community composition and temporal dynamics between the fractionated and de‐fractionated sets.

## Graphical abstract

Size fractionation differentiates large, particle‐associated microbes from small, free‐living ones. We show that pooling size‐fractionated marine samples, normalised by their DNA concentration, either before or after sequencing produces community profiles nearly identical to unfractionated samples. This provides a strong basis for comparing samples obtained from different filtration methods or different research projects. Size fractionation reveals niche‐specific associations that might otherwise be overlooked, enhancing our ability to detect interactions between small and large microorganisms during dynamic periods like spring blooms.

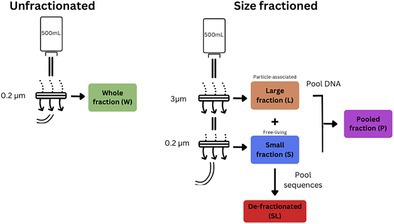

## 1 Introduction

Communities of microorganisms composed of bacteria, archaea, and unicellular eukaryotes dominate virtually all environments on Earth, from the depths of the ocean to complex terrestrial ecosystems. In the ocean, the heterogeneity of microbes attributable to the range of morphological, physiological, metabolic and behavioural differences grants essential and diverse ecosystem services, such as their contribution to the biogeochemical cycling of carbon and nutrients (Wong et al. 2015; Falkowski et al. 2004). Profiling of ocean microbiome diversity is routinely performed by sequencing a single target gene, commonly the universally present 16S ribosomal RNA (rRNA) gene for prokaryotes or the 18S rRNA gene for eukaryotes, at a given sampling location with protocols that target all microorganisms of a size greater than 0.2 μm.

Marker‐gene‐based profiling of the marine microbiome is carried out by filtering a known volume of seawater through a filter of a given pore size to collect cells. This is followed by DNA extraction from the particulate material collected on the filter and partial or complete sequencing of universal gene markers such as 16S rRNA or 18S rRNA. Size fractionation during the filtration step is a common practise in field sampling that is used to either increase the volume of water filtered through very small pore size (e.g., 0.2 μm) or to target specific groups of larger microorganisms (e.g., > 3 μm). Size fractionation increases labour and costs associated with marine microbiome research, but provides enhanced recovery of organism diversity with insights on the ecological functioning, trophic dynamics, growth and metabolic rates often associated with various size characteristics (Pommier et al. 2008; Massana et al. 2015; Benedetti et al. 2021; Kavagutti et al. 2023). Fraction‐specific taxonomic profiles can differentiate between same‐species life‐cycle stages, free‐living and particle‐associated prokaryotes, and pico‐, nano‐, micro‐, and meso‐plankton (Pascoal et al. 2023; Tao et al. 2022; Mestre et al. 2018). Microbes of different sizes in ocean microbiomes occupy distinct ecological niches, and contribute uniquely to ecosystem services (Finlay 2002; Wu et al. 2017; Tao et al. 2022).

The Bedford Basin is a semi‐enclosed 71 m deep bay located in the northwestern part of the Halifax Harbour, on the Atlantic coast of Nova Scotia (Raes et al. 2022; Robicheau et al. 2022, 2023). The surface waters have limited freshwater input, and a narrow sill connecting it to the open Atlantic Ocean leads to a stratified water column with saltier deep water, and fresh surface water (Li and Dickie 2001; Rakshit et al. 2023). During spring, increasing sunlight and warming surface water temperature induce stratification of the water column, triggering the spring bloom in the Bedford Basin. The bloom is dominated by a few species of phytoplankton that lead to a strong decrease in alpha diversity compared to pre‐bloom winter conditions. While size fractionation during a bloom period may improve the resolution of identified microbial species, the labour and cost‐intensive nature of size fractionation led us to assess the differences between size‐fractionated and unfractionated samples.

In this study, we explore the effects of using size fractionation with sequential 3 μm and 0.2 μm pore size filters and single filtration capturing particles > 0.2 μm. We evaluated how de‐fractionation, which we define as the reconstitution of the two size fractions into a single merged fraction, can accurately compare with unfractionated samples, and we suggest that size fractionation is advantageous during transitional periods, for example during a spring bloom. We used sequence reads from a universal primer targeting both 16S and 18S rRNA genes (Parada et al. 2016; Walters et al. 2016) in weekly seawater samples collected before, during and after the spring bloom of 2022 in the Bedford Basin. This sampling effort is part of a larger sampling effort of the Bedford Basin Monitoring Program. DNA sequences from fractionated and unfractionated samples were assigned to prokaryotic, chloroplast, and eukaryotic taxonomic groups. We focused on two approaches of sample reconciliation: combining the size fractions in proportion to their DNA concentrations relative to the total DNA of both fractions, or combining DNA extracts from the two size fractions in equal volume, prior to sequencing. We show that both of these reconciliation methods have high similarity to unfractionated samples, and that size fractionation is most useful for describing community members specifically by their size. Both approaches were thus considered effective in comparing water samples that were collected in parallel with and without size fractionation with discrepancies only observed in very rare ASVs.

## 2 Materials and Methods

### 2.1 Sampling and Environmental Data

For 16 weeks between January and April 2022, weekly water samples were collected using Niskin bottles at depths of 1, 5, 10, 30, and 60 m at the Compass Buoy Station in the Bedford Basin, Halifax, Nova Scotia, Canada (44.6936N, 63.6403W) with the exception of week 1 at 30 m which was not sampled. 500 mL of seawater from each depth was passed either sequentially through 3 and 0.2 μm filters (size‐fractionated) or directly onto 0.2 μm (unfractionated) 47 mm polycarbonate membranes (Millipore) using a peristaltic pump. Filters were flash frozen in liquid nitrogen and maintained at −80°C until DNA extraction. To prevent cross‐contamination, the four filtration lines are cleaned after each sampling day, and the samples are kept in a −80°C freezer, which is deemed to be best practise for long‐term storage (Bahl et al. 2012). Nutrient concentrations such as phosphate, silicate, nitrate, and ammonia, alongside chlorophyll a, and temperature data were provided by the Bedford Basin Monitoring Program (https://www.bio.gc.ca/science/monitoring‐monitorage/bbmp‐pobb/bbmp‐pobb‐en.php).

### 2.2 Extraction and Sequencing

DNA extraction and sequencing followed the protocols described in Robicheau et al. (2022). DNA was extracted from filtered biomass using the DNeasy Plant Mini kit (Qiagen) with an enhanced lysis procedure. Filters were incubated for 5 min at room temperature with 50 μL of 5 mg/mL lysozyme (Fisher BioReagents), then with 45 μL of 20 mg/mL proteinase K (Fisher BioReagents) and 400 μL of Buffer AP1 at 52°C for 1 h with continuous shaking in a ThermoMixer (Eppendorf). Subsequent extraction procedures followed the manufacturer's protocol; DNA was eluted in 100 μL of Buffer AE, and the concentration measured with a NanoDrop 2000c (Thermo Scientific). The V4–V5 region of the 16S rRNA gene, and V4 region of the 18S rRNA were sequenced on an Illumina MiSeq at the Integrated Microbiome Resource (Dalhousie University, Halifax, Nova Scotia, Canada) using the universal primers 515FB‐GTGYCAGCMGCCGCGGTAA and 926R‐CCGYCAATTYMTTTRAGTTT (Parada et al. 2016; Walters et al. 2016).

### 2.3 Data Processing

Sequence data was split into 16S rRNA and 18S rRNA amplicons using *bbsplit* (BBMap suite: Bushnell 2014) to bin reads with their respective target region using SILVA (Quast et al. 2012) and for the remainder of the analyses the 16S and 18S rRNA regions were processed separately. Unlike the 16S rRNA 390 bp target region, the 18S rRNA target region is longer (575–595 bp), yielding non‐overlapping paired reads. As a consequence, the forward and reverse reads were concatenated following the approach of McNichol (2019). 18S rRNA data was pre‐processed using packages within *bbmap‐env*: *bbduk* for trimming, and *fuse* for merging forward and reverse reads (Bushnell 2014). In contrast, 16S reads were trimmed and merged with DADA2 using a trimming setting of 280 nucleotides (forward) and 220 nucleotides (reverse). Both datasets were then denoised for amplicon sequence variant (ASV) using *DADA2* within QIIME2 (v. 2023.5.1) (Callahan et al. 2016; Bolyen et al. 2019). Chloroplast‐associated ASVs were removed from the 16S rRNA and re‐classified using *PhytoRef* (Decelle et al. 2015) for phytoplankton, and SILVA (Quast et al. 2012) was used for the prokaryote and eukaryote classification. Finally, we combined the ASV data with the metadata collected through the Bedford Basin Monitoring Program (Li and Dickie 2001).

### 2.4 De‐Fractionated Sets

To assess the effect of size fractionation on the characterisation of the microbial community, we categorised samples into four different size classes: small fraction (*S*, 0.2–3 μm), large fraction (*L*, > 3 μm), unfractionated whole (*W*, > 0.2 μm), and de‐fractionated small–large (*SL*, combined *S* and *L*). We refer to these by their acronyms in the manuscript. The *SL* fractions were generated by combining *S* and *L* sequences weighted by their *DNA* concentration according to Equation 1. Species that have less biomass in a sample tend to be under‐represented (Ficetola et al. 2008) and thus proportional with respect to *DNA* concentration merging is more representative of the > 0.2 μm sample. Pooling equally would have resulted in the over‐representation of the *DNA* from the > 3 μm fraction. Therefore, we normalised by the *DNA* concentration of each fraction to reflect the proportional difference in biomass yield between the fractions (Uhler et al. 2021; Elbrecht et al. 2017). By combining the fractions, we recapitulate the total diversity to estimate and compare with *W*. To calculate the ASV profile of *SL*, we combined the ASVs from each fraction (*S* or *L*), and calculated the relative abundance of ASV *i* in sample *j* (*C*_*ij*_) by normalising the observed relative abundances (*O*_*ij*_) with the total DNA concentration of the sample (Equation 1).

(1) $$C_{\text{ijSL}}=\frac{\left(O_{\mathrm{ijS}}\times {\left[\mathrm{DNA}\right]}_{\mathrm{jS}}\right)+\left(O_{\mathrm{ijL}}\times {\left[\mathrm{DNA}\right]}_{\mathrm{jL}}\right)}{{\left[\mathrm{DNA}\right]}_{T}}$$

where $O_{\mathrm{ijX}}$ is the observed relative abundance of ASV *i* in sample *j* from the size fraction *X*, $C_{\text{ijSL}}$ is the calculated relative abundance of ASV *i* in sample *j* from the size fraction *SL*, ${\mathrm{DNA}}_{\mathrm{jX}}$ is the *DNA* concentration of sample *j* from the size fraction *X*, and ${\mathrm{DNA}}_{T}$ is the sum of *DNA* concentration of sample *j* from the combined *L* (> 3 μm) and S (0.2–3 μm) fractions. While there were four fractions compared across all depths and weeks, a subset of 18 samples was selected for additional sequencing based on their Aitchison distance between the *SL* and *W* fractions (see Table S1 for highest and lowest Aitchison distances). We use Aitchison distance between the samples because it accounts for the compositional nature of sequencing data by performing a centred log‐ratio (CLR) transformation of the relative abundances (Quinn et al. 2018; Aitchison 1982). To assess whether the recombination in equal proportion from paired size fractions better represents the diversity recovered from unfractionated samples, we tested this approach using samples where *SL* was the most dissimilar (largest distance), or most similar (smallest distance) to *W*. We selected these samples to highlight how the pooling before sequencing may influence the distance to *W*, and to limit the sequencing cost of pooling all samples. This fraction will be referred to as pooled (*P*) since the DNA extracted from the *S* and *L* filters were pooled at a 1:1 volume ratio prior to sequencing. In contrast, *SL* was generated sequence reads obtained from the *S* and *L* fractions separately, and merged post‐sequencing using Equation (1).

### 2.5 Statistical Testing

Alpha‐diversity comparisons was assessed with richness counts on unrarefied data, and repeat rarefied data at different sequencing depths. The prokaryote‐derived reads were rarefied at 7500, chloroplast at 715 and eukaryotes at 50 for 100 iterations each, and the richness count at each iteration was averaged and reported. We selected these sequencing depths in order to retain at least 70% of reads. Since there is still debate around rarefaction for compositional data (McMurdie and Holmes 2014; Willis 2019; Weiss et al. 2017; Schloss 2024), we also included raw, unrarefied ASV counts because some of our > 3 μm samples have a low library coverage hence we would lose too many samples via rarefaction (see Figure S1 for rarefaction curves, Figure S2 for DNA concentrations). To compare the size fractions by their number of ASVs, we ran an ANOVA with post hoc pairwise *t*‐tests with Holm correction for *p*‐values. The richness was calculated as the total number of unique ASVs per sample, resulting in one richness value per week, depth, and size fraction. The null hypothesis of the *t*‐test is that the mean richness difference between pairs of fractions is zero. The result of the test is used as an indicator of the richness similarity between the size fractions. Since we have temporal data, we investigated abundance patterns over time through trend analyses, defined as the rate of community richness change over time as either increasing, decreasing, or constant. The trend was characterised through the slope of the linear regression of the richness with time. The time‐series analysis was used to assess whether the richness was stable across space, time and size fractions, and to evaluate the trend.

To quantify the overlap in ASV composition across size fractions, we defined sets for individual fractions, or for combination (union or intersection) of fractions. We then identified the number of shared ASVs between the fractions, and calculated their weighted contribution using the relative abundances of the ASVs using Equation 2. This weighted metric provides insights into whether the shared ASVs are rare or abundant.

(2) $$W_{s}=\frac{n_{s}\times \sum_{1}^{n}{\mathrm{RA}}_{\text{is}}}{\sum_{1}^{N}{\mathrm{RA}}_{i}}$$

where $W_{s}$ is the weighted proportion of a set *s*, and each set is a different size fraction. ${\mathrm{RA}}_{\text{is}}$ is the read count of a ASV *i* in set *s*, $n_{s}$ is the number of ASVs in the set *s*, *N* is the total number of ASVs in all sets, and ${\mathrm{RA}}_{i}$ is the read count of an ASV *i*. As a result, the weight of a set is the sum of the read counts of each ASV, multiplied by the ASV count in a set divided by the total read count of all ASVs in all sets. This analysis was conducted to compare the ASV composition between each size fraction.

We then conducted a differential abundance analysis to identify individual taxa that significantly differ in relative abundance between groups of size fractions *S*, *L*, *SL* and *W*. We used the Analysis of Composition of Microbiomes (ANCOM: Mandal et al. 2015) method from scikit‐bio to first normalise the read tables with CLR transformations appropriate for compositional data, then compare the relative abundance of each ASV pairwise across groups. ANCOM generates its own *W*‐statistic for each ASV which represents the number of times a test for this ASV was significantly different when tested against other ASVs. A high ANCOM W‐statistic suggests an ASV is highly differentially abundant between groups, making it an important ASV to distinguish between the groups which in this analysis are the different size fractions.

## 3 Results

### 3.1 Environmental Variation From January to April

**FIGURE 1:**
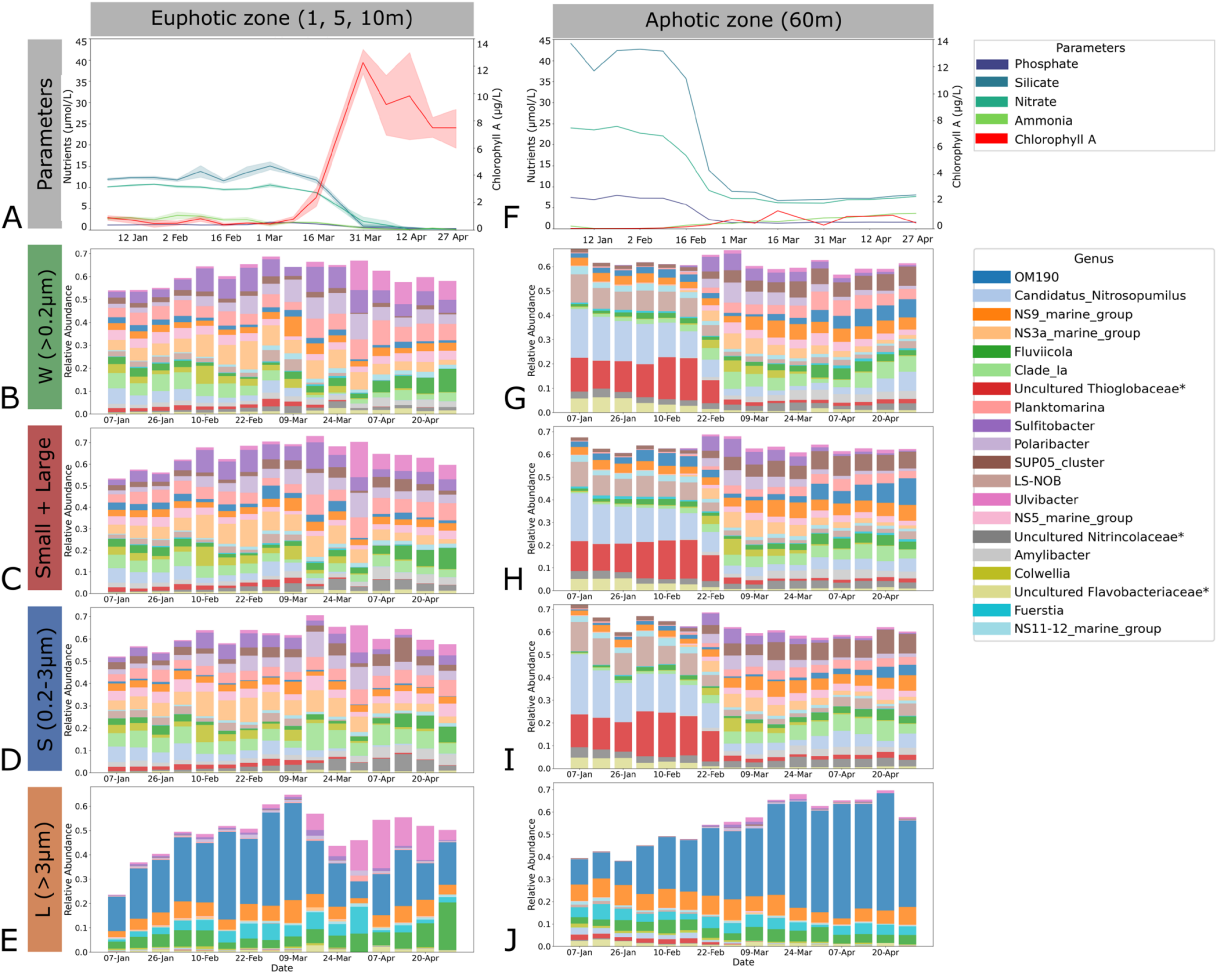
Concentrations of nutrients and chlorophyll a and prokaryotic microbial composition in the unfractionated (whole), de‐fractionated (*S* + *L*) and fractionated (*S*, *L*) sets. Bar plots of the concentrations of nutrients and chlorophyll a in the euphotic (1, 5 and 10 m) and aphotic zone (60 m) measured from January 7 to April 27, 2022. Nutrients including phosphate, silicate, nitrate and ammonia are quantified in µmol/L and chlorophyll a in μg/L. In the euphotic zone, solid lines represent the mean values across depths 1, 5 and 10 m and the shaded area indicates the 95% confidence intervals. Nutrient concentrations are represented by blue and green hues, and chlorophyll a in red. A secondary *y*‐axis was used for chlorophyll a to account for different scales facilitating the visualisation of its relationship with the nutrients. Below the nutrient plots, four taxonomic bar plots show the microbial composition in relative abundances for all non‐chloroplast 16S rRNA in the whole > 0.2 μm (B, G), de‐fractionated (C, H), small 0.2–3 μm (D, I) and large > 3 μm (E, J) size fractions during the spring bloom depicting the 20 most abundant genera. Some abundant genera were labelled as unclassified or uncultured. To provide more clarity, they were reidentified as uncultured or unclassified, along with their closest available taxonomic rank (e.g., genus, family, or class, depending on availability). These labels are also marked with an asterisk. The 5 m depth taxonomy was used for representing the euphotic zone. The taxonomic bar plots for chloroplast and eukaryotes are shown in Figures S1 and S4, respectively.

The 16 weekly samples were taken from January to April 2022, covering surface (depth 1 m) water temperatures ranging from 0.7°C to 6.6°C. The coldest and warmest temperatures were observed on February 7 and April 27, respectively. The chlorophyll *a* (Chl*a*) fluorescence, which is used as a proxy for phytoplankton biomass, remained below 1.1 μg/L until week 8 (March 1), and thereafter, increased to a peak value of 12.3 μg/L at week 12 (March 12) (Figure 1). The concurrent drastic drawdown of macronutrient concentrations (silicate, nitrate, phosphate, and ammonia), observed at week 12 (March 31) is also characteristic of coastal spring bloom dynamics (Robicheau et al. 2023). Due to the notable changes, we described weeks 1–8 (January 1 to March 1), and 9–16 (March 9 to April 27) as pre‐bloom and bloom periods, respectively.

In surface waters, we observe the shift from diverse communities to a few plastid ASVs, representative of the dominant phytoplankton species. The phytoplankton community shifted from a dominance of *Teleaulax*, *Bathycoccus*, *Thalassiosira* and *Micromonas* during the pre‐bloom period to free‐living *Pseudopinella* and Dictyophyceae or particle‐associated *Chaetoceros* during the bloom. These changes are observable in both the W and SL sets. Bacteroidota genera such as *Ulvibacter*, *Fluviicola* (detected in *L*) and *Polaribacter* increased in relative abundance in response to the bloom (see Supplementary Figure S4 for chloroplast taxonomic bar plots). OM190 was classified as the most abundant in *L* and its relative abundance increased in the aphotic zone during the bloom. This pattern is evident in the unfractionated samples, but the pattern is most prominent within the large fraction (Figure 1E,J).

Despite the overlap between detected ASVs in *SL* and *W*, the size‐fractionated samples allow for the distinction between free‐living and particle‐associated members. For example, the uncultured SUP05 clade Thioglobaceae is detected in *S*, *W* and *SL*, but size‐fractionated sampling reveals a clear dominance of this group in the small size fraction in the aphotic zone (60 m depth) during the spring bloom. Blast searches with the three ASV sequences assigned to the Thioglobaceae were 100% identical to *Candidatus Thioglobus autotrophica* (Shah and Morris 2015), *Pseudothioglobus* (Sadler et al. 2025) and to environmental sequences recovered from oxygen minimum zones (Walsh et al. 2009), respectively, pointing to a potential chemoautotrophic lifestyle. However, members of this clade are metabolically diverse (Morris and Spietz 2022), and we cannot determine based on 16S rRNA ASV sequences alone whether the SUP05 members recovered here from the deep water of the Bedford Basin are chemoautotrophs or heterotrophs (Walsh et al. 2009; Raes et al. 2022). In addition, the changes in relative abundance of *Sulfitobacter*, associated with sulphur cycling, and other heterotrophic taxa linked to the oceanic carbon cycle such as *Planktomarina* and Clade 1a (SAR11) (Marques et al. 2020; Gutiérrez‐Barral et al. 2021) are more noticeable in *S* and *L* fractions than in *W* (Figure 1). However, we can not confirm whether the ASVs detected in this study are purely heterotrophic members of these clades without additional genomic or metagenomic sequencing.

**FIGURE 2:**
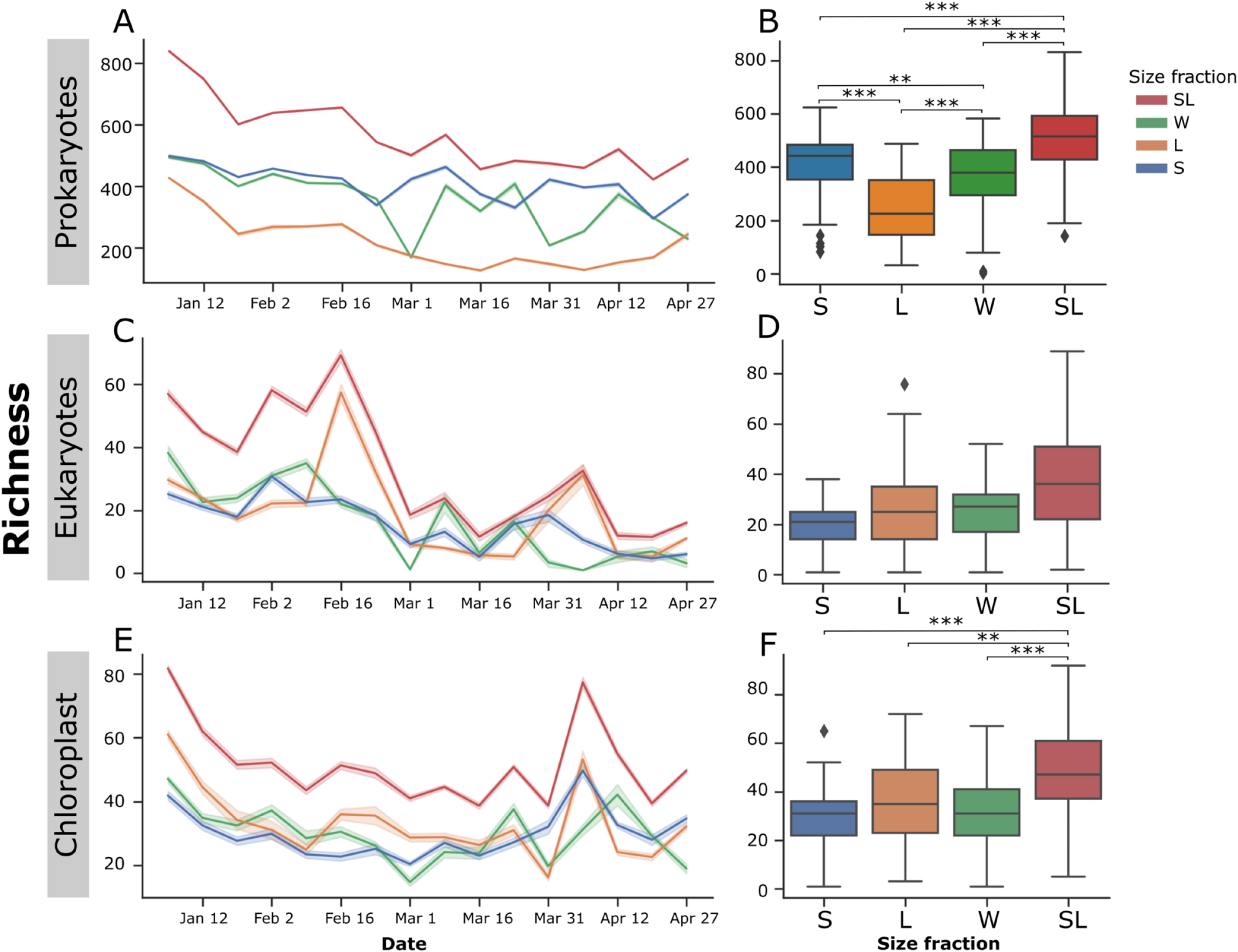
Repeat‐rarefied temporal dynamics of ASV counts for prokaryotes, eukaryotes, and chloroplast in the unfractionated, fractionated, and de‐fractionated samples. Line plots of the rarefied ASV richness (*y*‐axis) through time (*x*‐axis) for four size fractions for the 16S rRNA (A), 18S rRNA (C), and chloroplast (E) communities across 5 depths. The boxplots (B, D, F) show the richness (*y*‐axis) for each size fraction (*x*‐axis) across all weeks and depths. The median is represented as the line in the middle of the box, and the 25th, and 75th percentiles are the interquartile range (IQR). The whiskers are 1.5 times the IQR, and any outlier is a diamond outside the whiskers.

During the first 2 weeks of the bloom period, multiple unfractionated samples yield very low 18S rRNA reads but reconstituting *L* with *S* provided a more complete characterisation of the eukaryotes (Figure S3). Overall, size fractionation provides better resolution for prokaryotes, eukaryotes and chloroplasts, specifically during the bloom, both in the surface, and deep waters and allows for differentiation of dominant ASVs by size (Figure 2).

### 3.2 Small Prokaryotes Dominate Sample Richness

Prokaryotes represent 86.5%, chloroplast 12.7%, and eukaryotes 0.8% of the entire sequence library, i.e., all size fractions combined (Table 1). Across all size fractions, prokaryotes were preferentially amplified, and *SL* consistently had the highest richness. On average, the number of unique ASVs observed per sample is smallest for microbial eukaryotes (25±15 ASVs/sample), slightly higher for chloroplast (33±14 ASVs/sample), and highest for prokaryotes (366±132 ASVs/sample) independent of the size fraction (Figure S5). This difference in library size is partly due to the dominance of prokaryotes in pelagic environments, primer mismatches reducing the amplification of eukaryotic 18S rRNA, and the contribution of mitochondrial 16S rRNA of eukaryotes to the prokaryotic reads (McNichol et al. 2021). Paired *t*‐tests at each depth revealed no significant difference between the raw richness *S*, *L* and *W* size fractions for chloroplasts (*R*_*S*_ = 31, *R*_*L*_ = 35, *R*_*W*_ = 31) and eukaryotes (*R*_*S*_ = 19, *R*_*L*_ = 28, *R*_*W*_ = 26), but significant differences were observed when compared with *SL* (*R*_*SL*_ = 49 for chloroplast, *R*_*SL*_ = 37 for eukaryotes) (see Table S2 for all pairwise comparisons). The higher richness of the *SL* samples is due to the higher sequencing depth of combining two separately sequenced samples. Once rarefied, they yield similar richness across all fractions, suggesting that size fractionation does not capture more ASVs, and that *W* and *SL* have comparable richness counts.

**TABLE 1:** Read depth and number of ASVs observed in each size fraction for prokaryotes, chloroplasts and eukaryotes.

Community	Size fraction	Number of samples	Read depth	Number of ASVs
Prokaryotes	*L*	79	650,987	3048
	*S*	79	2,703,318	3223
	*W*	79	1,576,180	2935
	*SL*	79	4,451,599	6120
	*P*	18	1,295,732	963
Chloroplasts	*L*	79	303,259	226
	*S*	79	246,192	184
	*W*	79	174,940	188
	*SL*	79	583,130	333
	*P*	18	14,863	95
Eukaryotes	*L*	79	20,537	346
	*S*	79	9362	256
	*W*	79	12,889	284
	*SL*	79	22,917	588
	*P*	18	1922	117

*Note:* Breakdown of the number of ASVs and read depth for each sample type. Prokaryotes and eukaryotes are separated using SILVA as a reference, and chloroplasts are extracted from prokaryotic samples identified as ‘chloroplast’. Due to the overlap of ASVs between small (*S*) and large (*L*), the reconstituted small and large fraction (*SL*) is not the exact sum of ASVs between *S* and *L*. The pooled prior to sequencing fraction (*P*) method was only applied to 18 samples which were selected according to the Aitchison distance of samples reconstituted (*SL*) against unfractionated (*W*). The number of ASVs is aggregated from all depths, not averaged per depth.

### 3.3 Temporal Dynamics Vary More by Depth Than by Size Fraction

We compared the slope of richness change over time between size fractions to demonstrate alpha‐ diversity temporal dynamics. The temporal variation remained similar for all fractions, and they were always the same between the de‐fractionated (*SL*) and unfractionated (*W*) samples (see Figure S6). Characteristic of spring blooms, the bacterial richness in the upper (1, 5, 10 m) and mid (30 m) zones decreased from January to April, except for the small size fraction in the deepest (60 m) zone which increased slightly in richness (slope *S*_60 m_ = 0.023, Figure S6A–E). Chloroplast‐associated richness also decreased in the upper zone, and increased at 30 and 60 m as the bloom progressed. Eukaryotes from all depths decreased in richness from January to April (Figure S6F–J). The slope and trends between S and L varied for certain depth and community combinations highlighting the difference in richness between free‐living and particle‐associated members of the community throughout the spring bloom. For example, particle‐associated chloroplasts increased in richness in the aphotic zone but decreased or remained stable along the water column (Figure 6K–O). However, *SL* and *W* shared the same trend across depths, proving their comparability to track richness over time.

### 3.4 Similarity Among Samples Is Strongly Associated With Size Fractionation and Bloom Time

**FIGURE 3:**
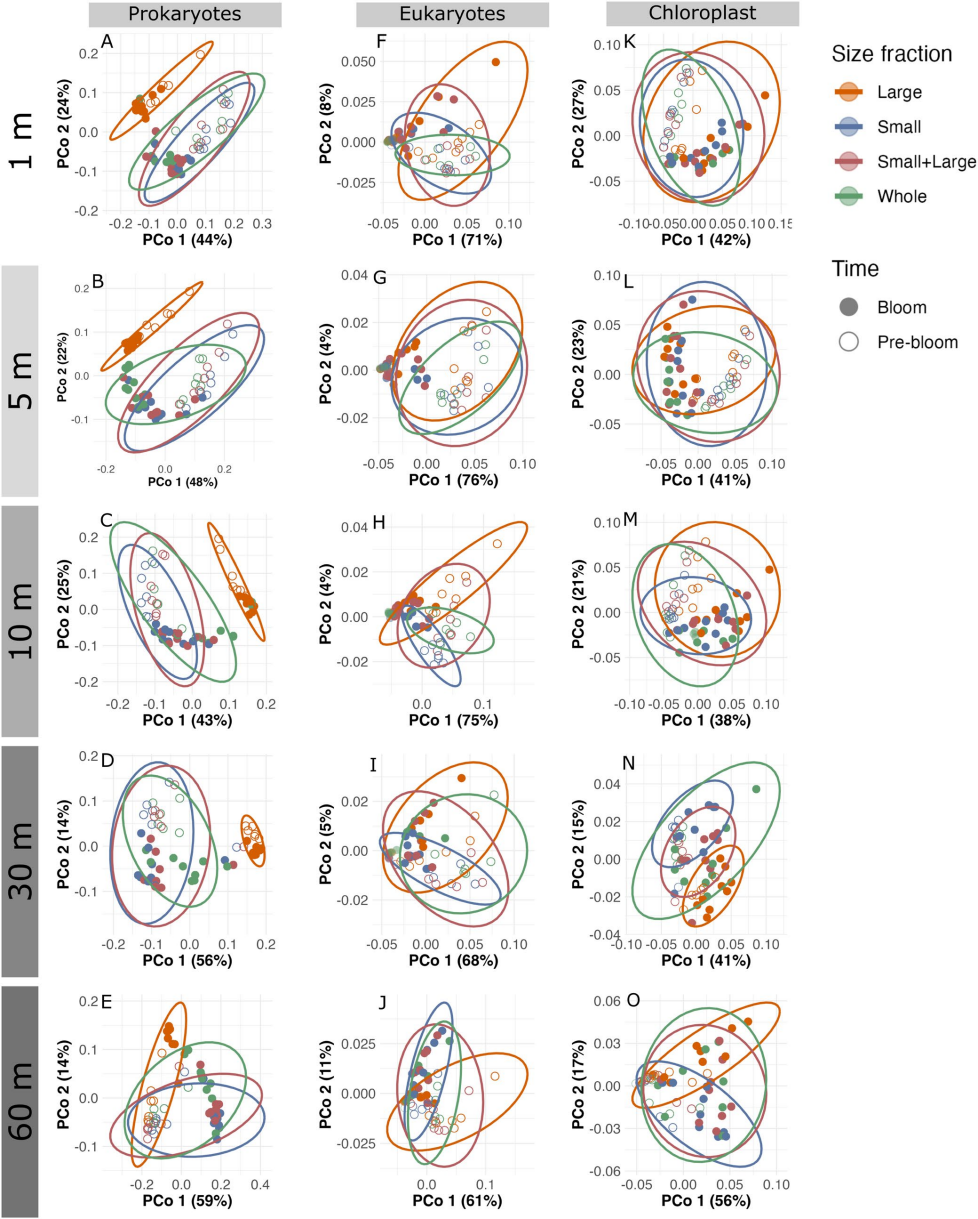
Temporal dynamics of ASV counts for prokaryotes, eukaryotes, and chloroplast in the unfractionated, fractionated, and de‐fractionated samples. Aitchison distances are displayed in scatter plots for 16S rRNA (left column), chloroplast (middle column) and 18S rRNA (right column). In each PCoA, one point represents a single sample, and the point fill represents the time. The pre‐bloom period (open dot) is from weeks 1 to 8, representing the period from January 7 to February 22, and the bloom period (filled dot) is from March 1 to April 27. The colours represent the size fraction, and confidence ellipses were drawn around each fraction in its respective colour where the small is in blue, large in orange, whole in green, and de‐fractionated fraction is red.

Beta‐diversity analyses supported by PERMANOVA testing confirmed the similar community composition of bacterial *S*, *W*, and *SL*, whereas *L* formed its own cluster. Pairwise PERMANOVA tests confirmed that the three fractions *S*, *W*, and *SL* have minimal variance between each other through large *p*‐values across the five depths, and small *R* ^2^ values (Figure 3A, Table S3). For eukaryotes and chloroplast, size fractionation did not result in a clear separation between size fractions, rather they formed temporal clusters that separated the pre‐bloom and bloom periods (Figure 3, Table S3). Our results support the notion that fractionation provides a distinct *L* fraction, but that *SL* samples are highly similar to *W* samples, and that the communities before and during the bloom are different.

### 3.5 Most Abundant ASVs Are Shared Between Size Fractions

**FIGURE 4:**
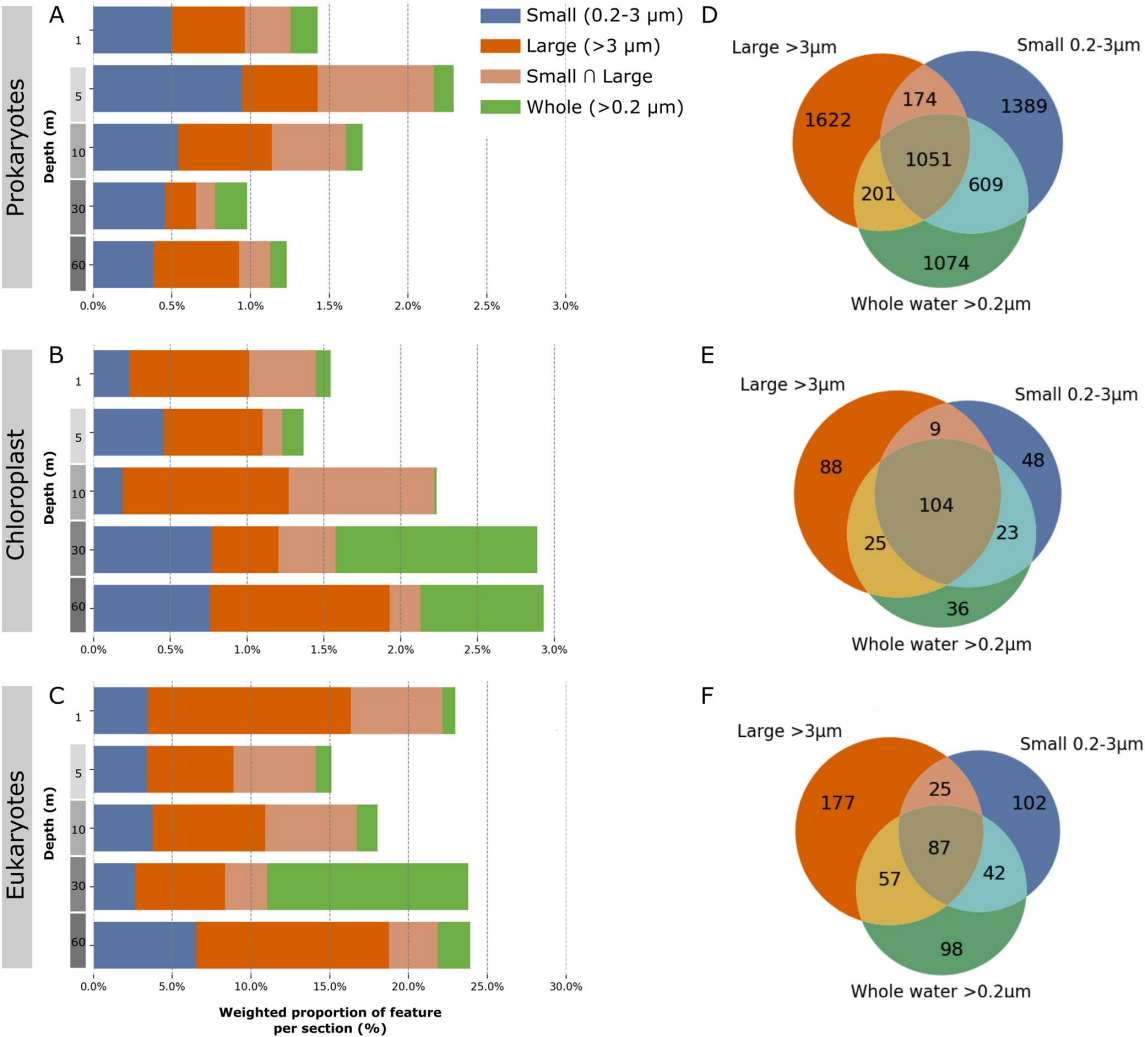
Multi‐depth ASVs overlap between unfractionated, fractionated and de‐fractionated prokaryotic, eukaryotic and chloroplast samples. Weighted (A–C) and unweighted (D–F) proportions of unique ASVs for prokaryotes (A, D), chloroplasts (B, E), and 18S rRNA (C, F). The *x* axis of the horizontal bar plots is truncated to improve the resolution of the weighted proportion unique to each set. Venn diagrams of the number of shared ASVs between sets of size fractions for (D) 16S rRNA, (E) chloroplasts, and (F) 18S rRNA amplicons. The colours are shared between the right and left plots where the small (0.2–3 μm) size fraction is in blue, the large (> 3 μm) size fraction in orange, the shared ASVs between size and large fraction in light orange, and the whole (> 0.2 μm) fraction in green. The brown section, the intersection of all three fractions ($W\cap \left(S\cup L\right)$), represents the ASVs that are commonly found in at least one of the size fractions, and *W*.

The majority of abundant ASVs are shared between *S*, *SL* and *W*, and only ASVs that contribute minimally to the overall composition are unshared. Our results show that 70%, 54% and 68% of ASVs are unique to individual size fractions for prokaryotes, chloroplasts and eukaryotes respectively (Figure 4D–F). However, these unique ASVs are very rare (Figure S8) and account for less than 3% of the total abundance for prokaryotes and chloroplasts, and less than 25% of microbial eukaryotes (Figure 4A–C). When weighted by abundance, we observe that most of the abundant taxonomic groups such as Alphaproteobacteria, Bacteroides, Gammaproteobacteria, *Teleaulax*, Dinophyceae, Syndiniales and *Thalassiosira* are shared in all size fractions.

### 3.6 Community Composition Difference Increases During the Spring Bloom

**FIGURE 5:**
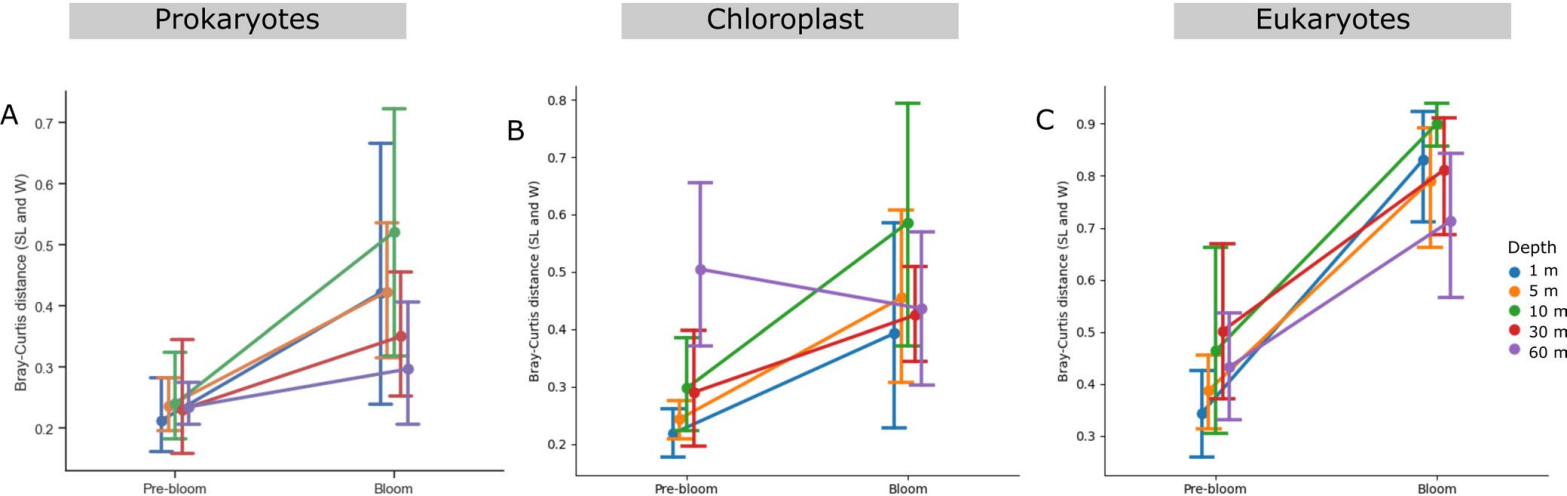
Bray‐Curtis distance between de‐fractionated and unfractionated samples before, and during the spring bloom at five depths. Mean Bray‐Curtis dissimilarity between size‐fractionated (*SL*) and unfractionated (*W*) samples at pre‐bloom and bloom periods. Each point represents the stage‐specific mean distance, with whiskers representing the 95% confidence interval. The slope represents the change in Bray–Curtis dissimilarity between SL and W samples from pre‐bloom to bloom. The colours represent the sampling depths in the Bedford Basin.

We characterised community composition before and during the bloom to assess whether *SL* similarity to *W* changed throughout the time series. Each sample had a most‐abundant genus we defined as “dominant”. A total of 16 distinct dominant genera were identified in the prokaryotic communities, 10 in the chloroplast communities and 46 in the eukaryotic communities (Figure S7). These patterns confirm that eukaryotes amplified with the universal primer have greater spatio‐temporal variability in taxonomic composition and fractions may be more difficult to reconcile. In contrast, bacterial *W*, *SL*, and *S* shared the same dominant taxa 87% to 100% of the time, and there is less variability in the dominant genera in the pre‐bloom period for prokaryotes, eukaryotes and cyanobacteria (Figure S7). This observation was confirmed with whole community dissimilarity comparison where we show that the community composition of *SL* and *W* was more dissimilar during the bloom, than before the bloom (Figure 5). We show that *W*, *SL* and *S* have compatible patterns of dominant genera and whole community composition, but there is more variation during the spring bloom, and for the eukaryotes specifically.

### 3.7 Important ASVs for Size Fraction Distinction

To better understand differences in relative abundance patterns across size fractions, we used ANCOM and pairwise ANCOM tests. Pairwise comparisons between fractions identified very few significantly different ASVs, and the global ANCOM tests (across all fractions) detected more differences driven by the distinct composition of the *L* fraction. Consistent with the rest of our results, the prokaryote *L* size fraction had on average two to three orders of magnitude more differentially abundant ASVs in comparison to chloroplast, or 18S rRNA at any depth (Table S2 for all pairwise *p*‐values). The pairwise testing between *W*, *SL*, and *S* revealed between zero and seven different ASVs (Table S2). Particularly *SL*, and *W* had only 2 significantly different ASVs, one detected at 1 m, and one at 60 m. Comamonadaceae, a freshwater bacterial family from the surface > 3 μm size fraction, and *Moritella* are both entirely absent from *W*, but detected at > 0.002 relative abundances in the *L* fraction, hence their presence in *SL*. These resulsts suggest that while some compositional differences in the community exist, the variations observed are subtle, supporting the comparibility of our size fractionation recombination approach for analysing microbial composition.

### 3.8 Re‐Pooling Size Fractions Prior to Sequencing

**FIGURE 6:**
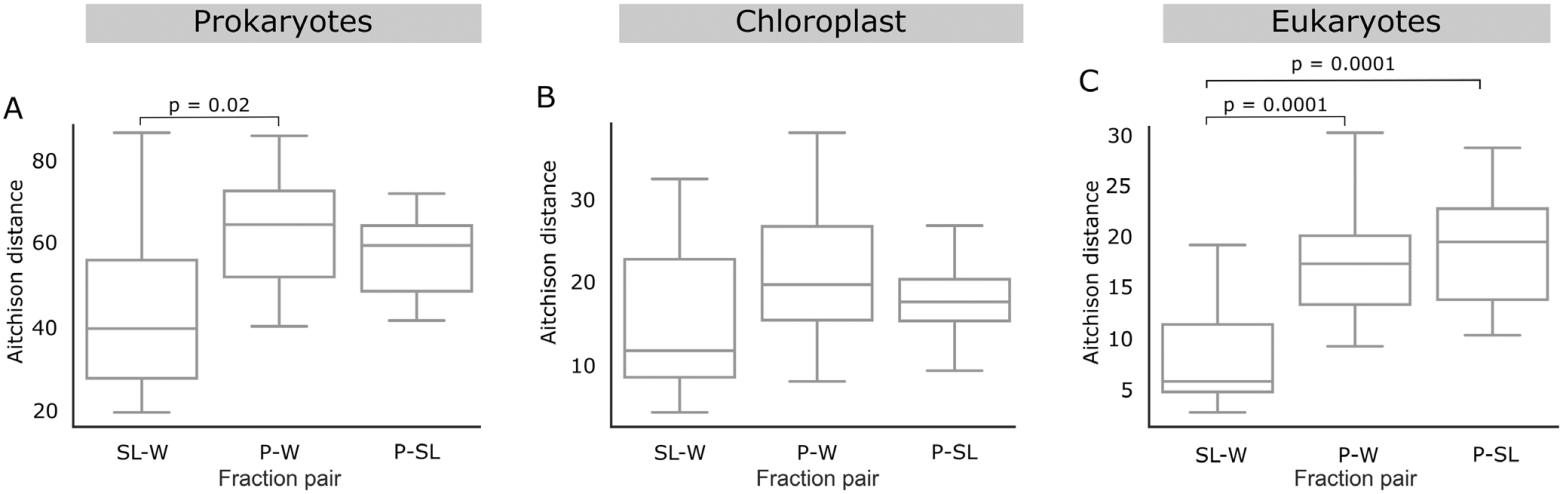
Aitchison distance between 18 pairs of samples that were unfractionated (*W*), fractionated and pooled prior to sequencing (*P*), or fractionated and pooled after sequencing (*SL*). Boxplot of the Aitchison distance between samples which were processed using different size‐fractionation protocols comparing the impact of pooling time (pre‐ or post‐sequencing). The boxplot shows the distribution of distances with smaller values indicating greater similarity. Post hoc *t*‐tests were conducted to assess whether the difference observed between the groups is significant, and significant *p*‐values (< 0.05) were reported on the plots.

To assess whether pooling size fractions before sequencing enhances the comparability to *W*, we selected 18 samples based on the Aitchinson distance to *W* (Table S1) to filter sequentially through 3 and 0.2 μm filters. We picked samples with poor and good representation to assess how pooling prior to sequencing might influence each. The resulting *S* and *L* fractions were pooled (*P*) on a 1:1 volume ratio. Consequently, *P* is a size‐fractionated sample that was sequenced and processed as a single unit, while *SL* consists of combined sequences from separately sequenced *S*, and *L* units. In essence, *P* underwent the same filtration process as *S* and *L* but was pooled before sequencing, resulting in a single sequencing step. *P* was not more similar to *W* than *SL* samples (Figure 6). The *SL* and *P* sets both showed a high degree of similarity to *W*, with no statistical difference seen in any of the communities examined (Figure 6). These results suggest that size‐fractionated samples can be merged prior to sequencing to save on sequencing costs and yield similar results.

## 4 Discussion

In this study, we investigated the impact of size fractionation on the composition and temporal dynamics of the Bedford Basin microbial communities. Our data span five depths over 16 weeks and we captured the diversity of microbial communities using a universal primer set that targets prokaryotes, eukaryotes and chloroplast marker genes. Our objective was to assess the similarities between microbial communities inferred from different filtration treatments and to determine how size fractions derived from small, or large pore sizes align with unfractionated samples and whether pooling fractions before or after sequencing impacts the similarity to unfractionated samples.

We found that dominant microbial ASVs are shared across the size‐fractionated (*S*, *L*) and unfractionated (*W*) sets. The de‐fractionated set where we combined *S* and *L* was very similar to *W* in terms of richness trends, dominant taxa and overall community composition suggesting that size fractionation does not significantly impact abundant community composition. However, the dissimilarity between *SL* and *W* increased during the spring bloom, suggesting that size fractionation is valuable during seasonal changes such as during blooms, intrusion events, or known environmental changes where larger cells or cells attached to particles may dominate the plankton community. Unsurprisingly, the *L* fraction was the most dissimilar to the unfractionated samples, suggesting that particle‐associated taxa have distinct community compositions that are highlighted only through size fractionation. However, pooling, either before or after sequencing of the *S* and *L* fractions yields communities comparable to those of unfractionated samples in terms of richness, beta‐diversity patterns, and dominant taxa. Some taxonomic groups such as OM_190 and Flavobacteriaceae exhibited different ASV‐specific time series highlighting how size fractionation can capture different changes in relative abundances which are sometimes inapparent or less obvious in unfractionated samples (Table 1).

### 4.1 Environmental Dynamics and Phytoplankton Blooms

Spring phytoplankton blooms typically form in late winter to early spring and are characterised by an increase in sunlight availability and temperature which stratifies the environment (Longhurst 1995; Li et al. 2006). The upper euphotic zone receives the most sunlight with decreasing light penetration as a function of depth. Phytoplankton species take advantage of these conditions for growth which leads to increased Chlorophyll *a* concentrations, and a rapid utilisation and depletion of nutrients (Dai et al. 2023; Needham and Fuhrman 2016). In our bacterial and chloroplast communities, the unfractionated (*W*) taxonomic profile of the 20 most abundant genera through the bloom is highly similar to that of the de‐fractionated (*SL*), for both the euphotic and aphotic zones. Our data reflect these seasonal changes with *W* and *SL* showing high taxonomic similarity during the bloom period but size fractionation highlighting distinct patterns of relative abundance changes between free‐living and particle‐associated microbes. Separating small and large fractions during the bloom prevents dominant biomass from monopolising sequencing depth, allowing us to recover both free‐living and particle‐associated prokaryotic, eukaryotic and chloroplast communities that are missed in the sequencing of unfractionated samples. The increased depth may also improve the resolution of rare members in low diversity conditions, and reveal size‐specific trends.

### 4.2 Impact of Size Fractionation on Inferred Community Composition

Our results show that fractionation using different pore size filters leads to different community compositions, specifically for particle‐associated members of the community. However, abundant taxa are shared between the fractions, suggesting the differences are driven by less abundant ASVs, particularly those with relative abundances ranging from 0 to 0.004 for prokaryotes, and 0 to 0.2 for eukaryotes and chloroplasts (Figure S8). *SL* has the highest richness, which is likely due to the higher sequencing coverage since *SL* represents two rather than one sample fraction. This is linked to an important methodological consideration that influences richness estimates and comparisons throughout our analysis of samples with different sequencing depths (Figure S2B). The particle‐associated community had lower richness in comparison to their free‐living counterpart only in prokaryotes, but not for chloroplasts and eukaryotes, a trend that was observed in both unrarefied and rarefied samples (Figure 2, Figure S5).

We used log‐ratio transformations (Gloor et al. 2017) as an alternative to rarefaction. The DNA concentrations were highest in the unfractionated samples, and peaking in mid‐April (weeks 14, 15, 16), while the lowest concentrations were consistently observed in the > 3 μm fraction (Figure S2A). The likelihood of consistently detecting rare ASVs across samples due to biological and sampling variability is small making it difficult to track temporal trends of rarer ASVs, specifically in low biomass samples. Previous studies showed that filtering out rare ASVs, including those of low abundance within and across samples, can improve reproducibility, and comparability of results (Schloss 2018; Cao et al. 2021). However, we chose to retain rare ASVs to provide a comprehensive comparison, as rare ASVs may play ecologically important roles. In separating the microbial community by size, we enhance the detection of rare ASVs by reducing the effect of abundant taxa, resulting in higher observed richness in comparison to unfractionated samples. We demonstrate that abundance patterns are well aligned between *W* and *SL* but tracking temporal patterns of individual ASVs can be misleading. Some ASVs are differentially represented across size fractions, and their time series are in consequence divergent in *S* and *L* (McLaren et al. 2019) (Figure S9). Therefore, while community‐level abundance patterns align well between *SL* and *W*, individual ASV time series and the sample absolute richness are different between size‐fractionated and unfractionated samples.

### 4.3 Ecological Relevance of Size Fractionation

According to our results, we suggest that size fractionation and merging provide two main advantages. First, it increases the recovery of rare taxa during bloom periods when the community diversity is lower by increasing the sequencing depth. Second, merging the fractions allows for direct comparison of fractionated, and unfractionated datasets, improving the interoperability across studies that use different sampling and sequencing approaches. Size fractionation may also help to resolve microbial groups based on their ecological roles or functional profiles based on size. Particle‐associated microbes are estimated to have different metabolic activity compared to free‐living ones and describing size‐specific microbial abundances may be more relevant during a spring bloom for processes associated with organic matter degradation (Wang et al. 2024; Lloyd et al. 2022). We effectively show that when the community is stable (pre‐bloom state), *SL* and *W* have very small Bray‐Curtis distances suggesting the communities are very similar but during the bloom period where changes occur, diversity decreases and there are changes in the community. In such cases, size‐fractionated data can provide more ecologically meaningful insights by better capturing the organisms involved in key processes such as microbial succession or nutrient utilization than unfractionated measures alone.

### 4.4 Methodological Considerations for Size Fractionation

While size fractionation can enhance microbial community representation due to a more refined community description by size, our findings suggest that the pooled fraction is similar in terms of taxonomic composition to the unfractionated samples. This is expected as relative abundances derived from amplicon sequencing exhibit higher variance than absolute presence‐absence counts of ASVs for cross‐sample comparisons, particularly when not paired with methods for absolute quantification (Clausen and Willis 2021; Nearing et al. 2021). Therefore, if interest is in rare ASVs, specifically divided by their physical size, size fractionation justifies the additional time, and budget allocation. In addition, the fractionation can provide insights into functionality since free‐living and particle‐associated microorganisms are associated with distinct niches in ocean microbiomes. However, for analyses focused on abundant lineages, we suggest only one filtration step for longitudinal amplicon analysis of microbial communities. In this study, we have used an extensively validated universal primer set to target both prokaryotic (16S), and eukaryotic (18S) rRNA genes (McNichol et al. 2025), but the majority of the reads in our dataset were of bacterial and plastid origin. This outcome likely results from the higher abundance of bacterial templates. In addition, the sequencing depth that was achieved with these samples may have reduced the recovery of 18S rRNA. As a consequence, the eukaryotic community profile is less comprehensive and more variable than that of the prokaryotes and we therefore interpret the eukaryotic results with caution. However, the low recovery of eukaryotes does not impact our conclusions on size fractionation and our approach to reconstitute fractionated and unfractionated samples.

## 5 Conclusion

We assessed the effect of sequential filtration through > 3, and > 0.2 μm pore size filters, with single‐step filtration on microbial community composition and diversity during a spring bloom in a Northwestern Atlantic microbial community. We show that pooling size‐fractionated samples by DNA proportion produces microbial community compositions determined by 16S rRNA amplicons highly similar to those of unfractionated samples which underwent a single filtration step. Our analysis reveals that the small size fraction closely resembles unfractionated samples, and the *L* size fraction consistently represents a distinct community. However, when *S* and *L* samples are pooled, the majority of abundant taxa are shared with the unfractionated samples, indicating that size fractionation does not necessarily provide additional insights into the dominant microbial communities. However, size fractionated samples have higher richness and provide insights into rare taxa that are poorly characterised in unfractionated samples. This enhanced representation of rare taxa is due to the inherent nature of compositional data product by high throughput sequencing, splitting, and separately sequencing samples creates more space to capture rare taxa that are otherwise obscured or undetected in unfractionated samples. We show that pooling fractions prior to or after sequencing does not linearly impact the data and thus yields highly similar results, and therefore is an appropriate method to compare unfractionated with fractionated samples.

## Author Contributions

**Diana Haider:** conceptualization, data curation, investigation, software, validation, formal analysis, visualization, writing – original draft, writing – review and editing, methodology, resources. **Jennifer Tolman:** conceptualization, methodology, resources, writing – review and editing, investigation. **Robert G. Beiko:** project administration, funding acquisition, writing – original draft, writing – review and editing, conceptualization, investigation, validation, supervision, resources. **Julie LaRoche:** project administration, funding acquisition, writing – original draft, writing – review and editing, conceptualization, investigation, validation, supervision, resources.

## Funding

This work was supported by Ocean Frontier Institute and Natural Sciences and Engineering Research Council of Canada.

## Conflicts of Interest

The authors declare no conflicts of interest.

## Supporting information


**Data S1:** emi470206‐sup‐0001‐Supinfo.pdf.

**Data S2:** emi470206‐sup‐0002‐Data.csv.

## Data Availability

The raw sequencing data that supports the findings of this study is openly available in the National Center for Biotechnology Information (NCBI) BioProject PRJNA785606. The scripts used to generate all the results are available on GitHub https://github.com/dianahaider/size_fractions and https://doi.org/10.5281/zenodo.17162071. The accompanying unmodified formatted metadata derived from the NCBI SRA Run Table (SraRunTable.csv) is available in the GitHub repository and in Supporting Informations S1 and S2, providing sample and experimental descriptions.
